# The *STF2p* Hydrophilin from *Saccharomyces cerevisiae* Is Required for Dehydration Stress Tolerance

**DOI:** 10.1371/journal.pone.0033324

**Published:** 2012-03-16

**Authors:** Gema López-Martínez, Boris Rodríguez-Porrata, Mar Margalef-Català, Ricardo Cordero-Otero

**Affiliations:** Department of Biochemistry and Biotechnology, University Rovira i Virgili, Tarragona, Spain; Institute of Developmental Biology and Cancer Research, France

## Abstract

The yeast *Saccharomyces cerevisiae* is able to overcome cell dehydration; cell metabolic activity is arrested during this period but restarts after rehydration. The yeast genes encoding hydrophilin proteins were characterised to determine their roles in the dehydration-resistant phenotype, and STF2p was found to be a hydrophilin that is essential for survival after the desiccation-rehydration process. Deletion of *STF2* promotes the production of reactive oxygen species and apoptotic cell death during stress conditions, whereas the overexpression of *STF2*, whose gene product localises to the cytoplasm, results in a reduction in ROS production upon oxidative stress as the result of the antioxidant capacity of the STF2p protein.

## Introduction

The kingdoms of bacteria, fungi and plants contain anhydrobiotic organisms that can survive during water-deficient periods [1]. During dehydration, the metabolic processes of these organisms are in a suspended state. However, during desiccation stress, genes are expressed that promote cellular tolerance to dehydration through protective functions in the cytoplasm, an alteration in the cellular water potential to promote water uptake, the control of ion accumulation, and the further regulation of gene expression [2]. Some of the proteins termed hydrophilins participate in the cellular tolerance to this stress condition and are biochemically characterised by a Gly content greater than 6%, a hydrophilicity index of >1.0, and a secondary structure of 50–80% coils. The genome of *Saccharomyces cerevisiae* contains genes (*GON7*, *GRE1*, *HSP12*, *NOP6*, *RPL42A*, *STF2*, *SIP18*, *TIF11*, *WWM1*, *YBR016w*, *YJL144w*, *and YNL190w*) that code for proteins with the characteristics of hydrophilins. The fact that the transcripts of all of these genes accumulate in response to osmotic stress suggests that the expression of hydrophilins represents a widespread adaptation to water deficit [3]. The properties of hydrophilins include their roles as antioxidants and as membrane and protein stabilisers during water stress, either by direct interaction or by acting as molecular shields [4]. Although the functional role of most *S. cerevisiae* hydrophilins remains speculative, there is evidence supporting their participation in the acclimation or adaptive response to stress [5]. The ectopic expression of some hydrophilins in yeast confers tolerance to water-deficit conditions [6], [7], [8], and the presence of these proteins has been associated with chilling tolerance [9].

In this study, we evaluated the role of the aforementioned *S. cerevisiae* hydrophilins in dehydration stress. Five strains overexpressing *SIP18*, *STF2*, *GRE1*, *YJL144w* or *NOP6* were identified as dehydration tolerant. For *STF2*, we found that the cell viability after desiccation and rehydration process was due to the antioxidant capacity of this protein, which reduced the number of apoptotic cells during stress conditions by minimising the accumulation of ROS in the cells.

## Results

### Hydrophilins from S. cerevisiae enhance dry stress tolerance

**Figure 1:**
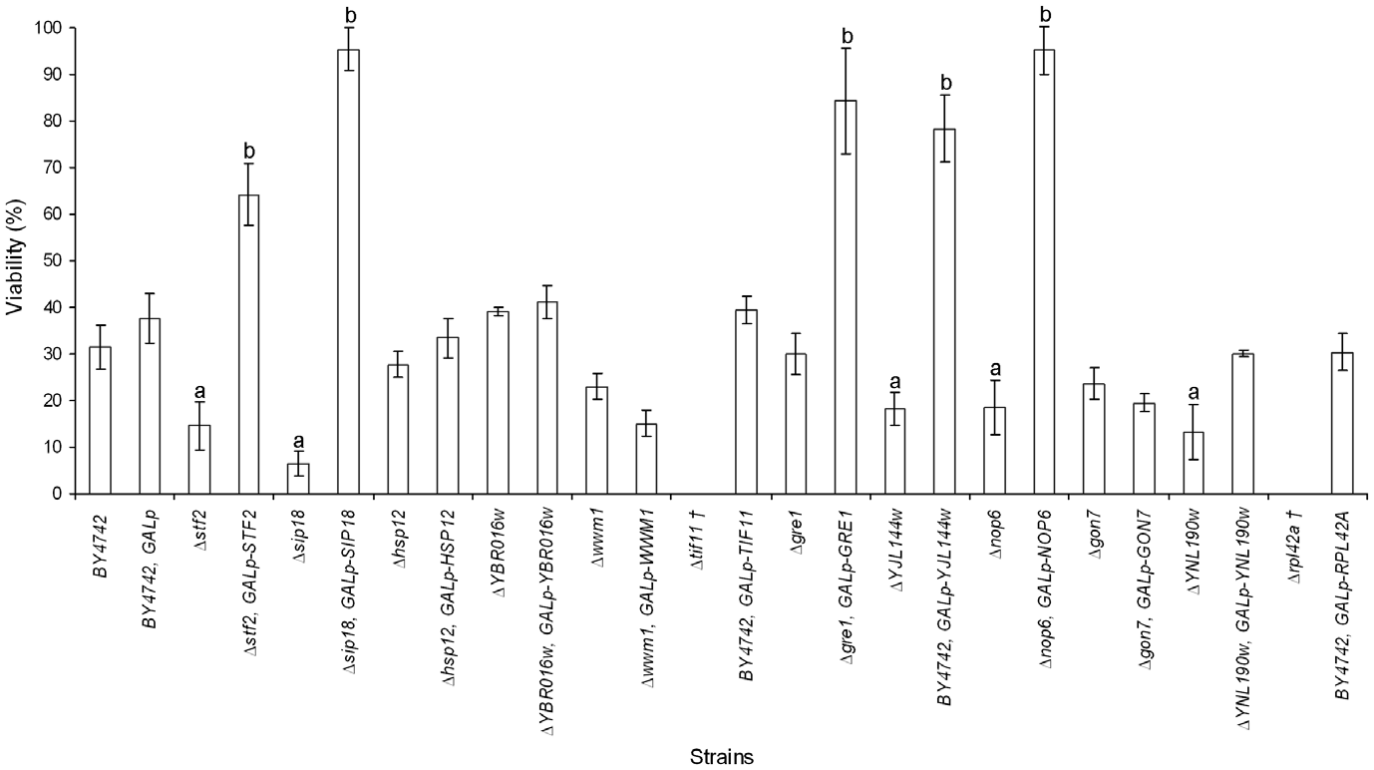
Effect of over-expressing hydrophilin genes on the yeast viability after the drying and rehydration process. The scale of viability (%) indicates the percentage of experimental values for the different strains. Values shown are the means of at least *n* = 3 independent samples ± the standard deviation. ^a,b^Significant differences (*p*≤0.05) with respect to the BY4742 and to the BY4742, *GALp* strain, respectively.

Among the 12 proteins of the hydrophilin group found in *S. cerevisiae*, TIF11p and RPL42Ap are encoded by essential genes. Therefore, the desiccation tolerance capacity of a set of 10 viable mutant haploid strains (BY4742) for the genes encoding these hydrophilins was assessed using a colony-counting assay. The mean CFU (colony-forming units) ml^−1^ value for survival after rehydration was calculated after taking into account the viability before drying. Only the Δ*stf2*, Δ*sip18*, Δ*YJL144w*, Δ*nop6* and Δ*ynl190w* strains (BY4742 background) exhibited statistically significant lower values of viability after stress induction, 14%, 6%, 18%, 18% and 13%, respectively, from the BY4742 strain (Fig. 1). The viability of the Δ*hsp12*, Δ*YBR016w*, Δ*wwm1*, Δ*gre1* and Δ*gon7* strains did not exhibit statistically significant differences from the reference strain (at ∼35%). We next characterised the effects of increasing the STF2p, SIP18p, HSP12p, YBR016wp, WWM1p, TIF11p, GRE1p, YJL144wp, NOP6p, GON7p, YNL190wp and RPL42ap expression levels in stationary-state cells using a plasmid that allows expression of these genes under the control of the *GAL1* promoter (*pGAL1*) in the corresponding yeast gene-deletion strain (except for the two essential genes, *TIF11* and *RPL42*, which were over-expressed in the BY4742 strain). After rehydration, the following strains exhibited approximately 50% higher viability than the BY4742, *GALp* strain: Δ*stf2*, *GALp-STF2*; Δ*sip18*, *GALp-SIP18*; Δ*gre1*, *GALp-GRE1*; Δ*YJL144w*, *GALp-YJL144w*; and Δ*nop6*, *GALp-NOP6* (Fig. 1). Furthermore, the other transformant strains showed cell viability values similar to that of the reference strain harbouring the empty vector (i.e., BY4742, *GALp*). These results allowed us to conclude that the *STF2*, *GRE1*, *NOP6*, *YNL190w*, *SIP18* and *YJL144w* genes -the last two having been the subject of a previous study [10]- are essential to overcome the simple stress of the desiccation-rehydration process. Moreover, the increased levels of *STF2*, *SIP18*, *GRE1*, *YJL144w* and *NOP6* gene products before stress induction might enhance the dehydration stress tolerance.

### Overexpression of STF2 prevents cellular ROS accumulation

**Figure 2:**
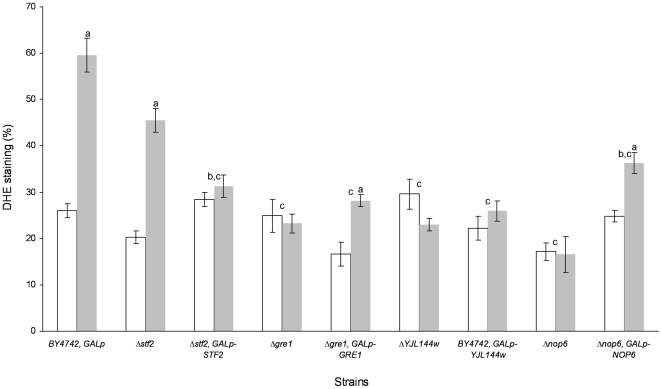
Yeast cells accumulate ROS during stress induction. Quantification of the ROS accumulation using DHE staining before cell dehydration (BD, white bars) and after the rehydration process (AR, grey bars). Values are the means of at least *n* = 3 determinations ± the SD. ^a^Significant differences (*p*≤0.05) during stress induction with respect to the BD step. ^b^Significant differences (*p*≤0.05) during stress induction of overexpressing strain with respect to the knock-out strain. ^c^Significant differences (*p*≤0.05) during stress induction with respect to the *BY4742*, *GALp* strain. In each experiment 500 cells were evaluated.

**Figure 3:**
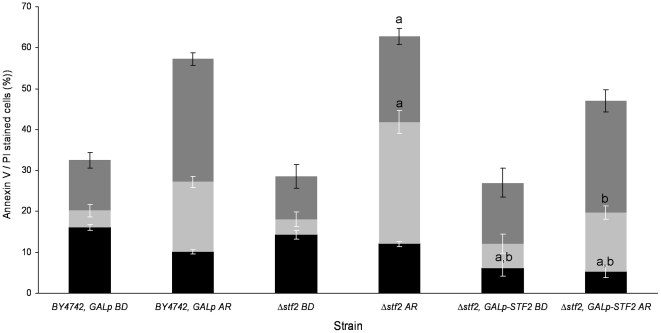
Apoptotic hallmarks in the STF2p-over-expressing strain. Stained cells: 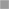, necrotic; 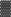, secondary necrotic; and ▪, apoptotic cells. The scale of Annexin V/PI-stained cells (%) indicates the percentage of experimental values for the different strains BD and AR. The represented values are the means of *n* = 3 determinations ± the SD. ^a,b^Significant differences (*p*≤0.05) with respect to BY4742, *GALp* and *Δstf2* strains, respectively. In each experiment, 1×10^5^ cells were evaluated.

Based on the reported antioxidant role of hydrophilins in different organisms, as reviewed by [4], we wanted to ascertain, in stationary-state cells, whether the higher viability rate of the STF2p, GRE1p, YJL144wp and NOP6p over-expressing strains relative to the wild type after the de- and rehydration process could be due to differences in ROS accumulation [11]. Yeast cells in the stationary phase and after rehydration were incubated in the presence of dihydroethidium (DHE) to quantify the ROS-accumulating cells (Fig. 2). Before dehydration, approximately 29% of the cells of each evaluated strain showed fluorescence after DHE incubation. After rehydration, the cultures of BY4742, *GALp* and Δ*stf2* strains contained up to 35% more cells that exhibited intense intracellular DHE staining. During stress induction, the Δ*stf2*, *GALp-STF2* cells (30%) showed a statistically significant reduction in ROS accumulation in comparison to the Δ*stf2* cells (45%). However, the Δ*gre1*, *GALp-GRE1*; Δ*YJL144w*, *GALp-YJL144w*; and Δ*nop6*, *GALp-NOP6* cells did not show statistically significant reduction in fluorescence in comparison to Δ*gre1*, Δ*YJL144w* and Δ*nop6* cells. Notably, around 32% of Δ*gre1*; Δ*gre1*, *GALp-GRE1*; Δ*YJL144w*; Δ*YJL144w*, *GALp-YJL144w*; Δ*nop6*; and Δ*nop6*, *GALp-NOP6* cells showed 50% lower ROS levels during stress induction than By4742, *GALp* cells. Considering the cell viability results for the over-expressing strains (Fig. 1) and their ROS reduction values in comparison to the corresponding knock-out strain (Fig. 2), we suggest that only STF2p overexpression correlates with the increase in the desiccation survival rate and the reduction in ROS levels after stress induction. Therefore, we explored whether the changes in the cell viability observed in the Δ*stf2*, *GALp-STF2* strain with elevated dehydration tolerance correlated with other apoptotic processes, such as phosphatidylserine externalisation (Annexin V/PI staining) and DNA strand breaks (TUNEL assay) (Fig. 3). Using flow cytometry, we were able to quantify apoptotic (Annexin V^+^/PI^−^), secondary necrotic (Annexin V^+^/PI^+^), and true necrotic (Annexin V^−^/PI^+^) cells. After stress induction, the Δ*stf2* strain showed amounts of apoptotic (∼12%, as BY4742, *GALp* strain) and secondary necrotic (∼29%) fluorescent cells significantly higher than Δ*stf2*, *GALp-STF2* cells, ∼5% and ∼15% respectively, whereas the reference strain and the dehydration-tolerant clone had similar percentages of Annexin V/PI and PI cells, ∼15% and ∼29%, respectively. Additionally, before dehydration, the percentages of Annexin V, Annexin V/PI and PI cells for the BY4742, *GALp*; and Δ*stf2* strains did not exhibit significant differences, with staining levels of ∼15% for Annexin V, ∼5% for Annexin V/PI and ∼11% for PI, respectively for both strains, whereas the Δ*stf2*, *GALp-STF2* strain showed similar percentages Annexin V/PI and PI cells but only 5% for Annexin V cells. These results suggest that the over-expression of STF2p minimised the number of apoptotic cells during stress induction as a putative consequence of the reduction of ROS accumulation in the cells. By the contrary, cell death of Δ*gre1*, Δ*YJL144w* and Δ*nop6* strains might be linked to some molecular pathway in a ROS accumulation-independent way.

### GFP-STF2 fusion protein accumulates in the cytoplasm

**Figure 4:**
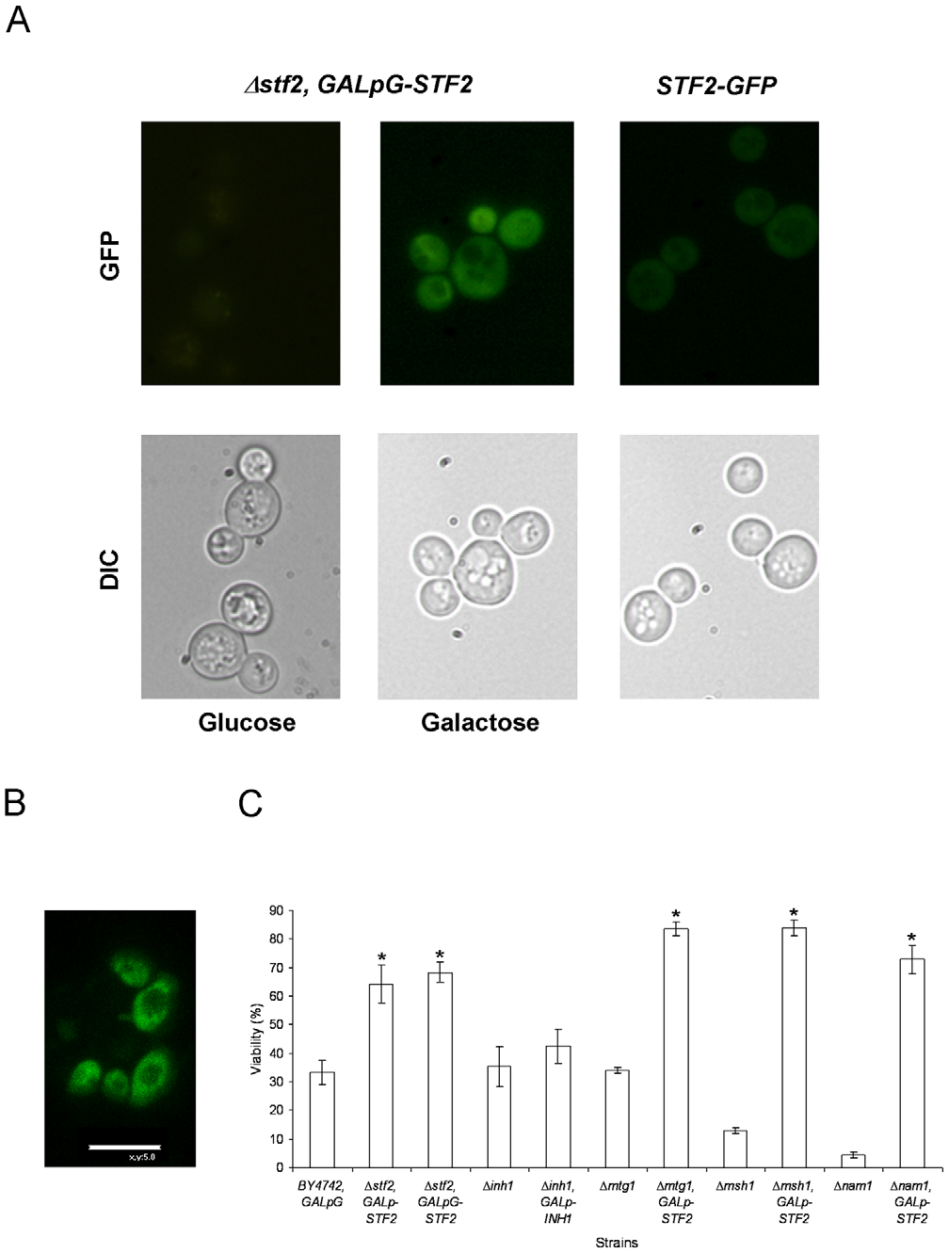
*STF2-SIP18* fusion localises to the cytoplasm. A) Each column shows images of the same field, with the fluorescence of the green fluorescent protein (GFP) in the top row and the differential interference contrast (DIC) images of the cultured yeast cells in the bottom row. The Δ*stf2* cells transformed with the vector expressing *GFP-STF2* under the control of the *GAL* promoter were photographed after 4 h of galactose or glucose supplementation. Cells expressing the GFP-STF2 fusion protein under the *STF2* promoter were photographed after 24 h in the stationary phase. B) Analysis of cells expressing GFP-STF2p using the confocal microscope. Image generated by the average of a pile of five optical sections. C) The scale of viability (%) indicates the percentage of the experimental values for the different strains after the dehydration and rehydration process relative to the highest value for the fresh cultures before the induction of the stress. Values are the means of *n* = 3 determinations ± the SD. *Significant differences (*p*≤0.01) of overexpressing strains with respect to the BY4742, *GALpG* strain.

With the aim of investigating the localisation of STF2p, a strain carrying a fusion of STF2p and green fluorescent protein (GFP) integrated in the *STF2* locus (*GFP-STF2*) was analysed by microscopy after 2 days of growth (*STF2* is mainly expressed during the stationary phase [12]. After 48 h of growth, a culture of the Δ*stf2* strain with the plasmid expressing *GFP-STF2* under *GALp* (Δ*stf2*, *GALpG-STF2*) was divided, and the stationary-state cells were observed after a 4 h supplementation with 2% galactose or 2% glucose. The fusion protein was expressed at a very low level in the presence of glucose, resulting in the diffuse labelling of the cells, mainly due to the low activity of *GALp* even after glucose starvation. However, the Δ*stf2*, *GALpG-STF2* cells supplemented with galactose exhibited a high fluorescent signal, with most cells exhibiting green fluorescence in the cytoplasm (Fig. 4A). In order to better note the localization of the full protein in the cytoplasm, cells of the over-expressing GFP-STF2p fusion strain were observed after 4 h of galactose induction, using a confocal microscope. Labelling of the cell surface, nucleus or vacuolar system was not observed in any case (Fig. 4B). Additionally, cells of both the Δ*stf2*, *GALpG-STF2* and Δ*stf2*, *GALp-STF2* strains (Fig. 4C) showed the same increase in viability after rehydration in comparison with the reference strains harbouring the empty vectors (Figs. 1 and 4C). Therefore, the GFP tag did not result in any phenotypic defect in the viability of the BY4742 strain after the dehydration and rehydration process.

### Respiration deficiency in the GALp-STF2 strain does not promote dehydration tolerance

**Figure 5:**
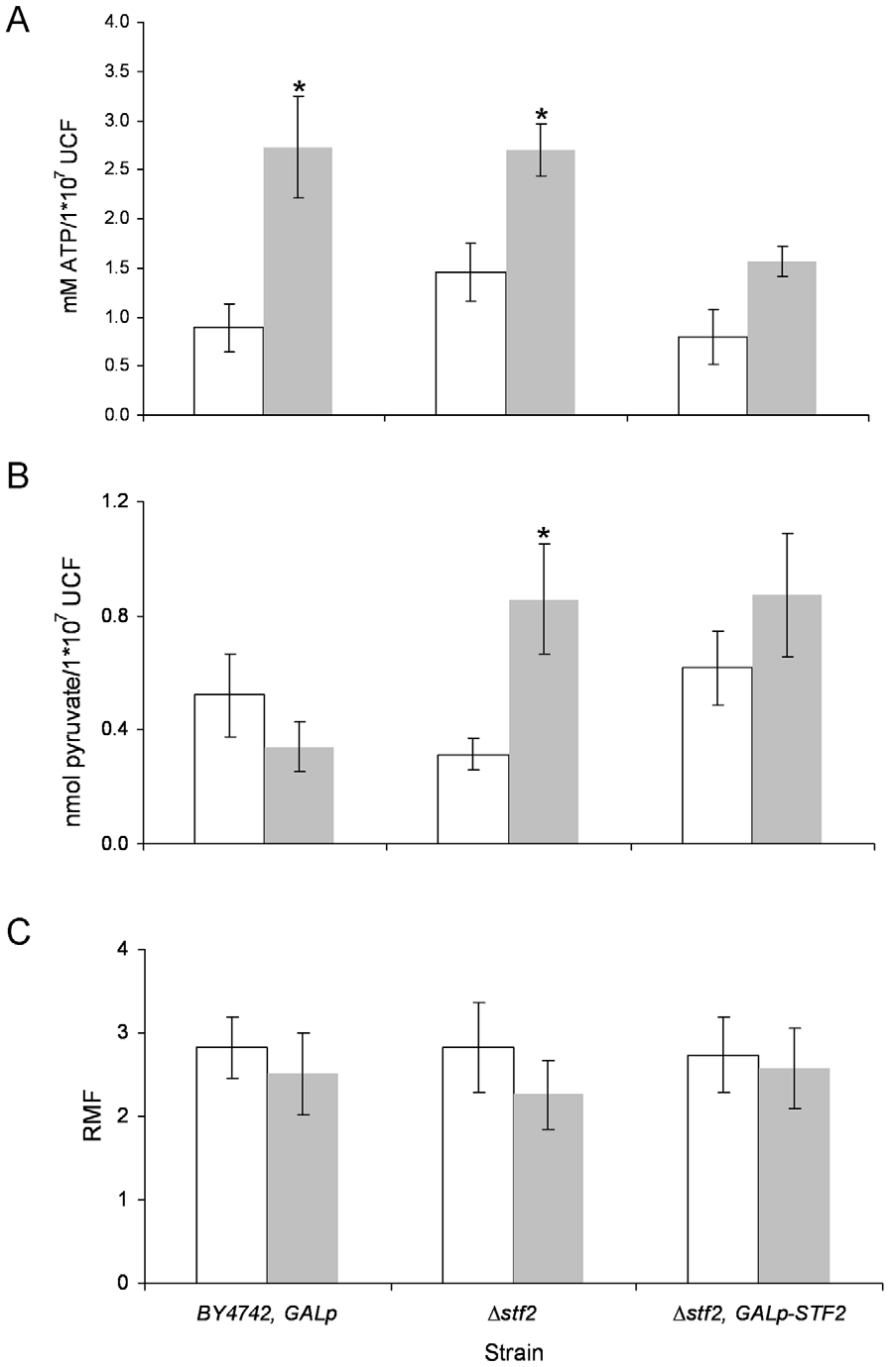
Quantification of ATP (A), pyruvate (B), and the relative mitochondrial function (C) BD (white bars), AR (grey bars) and relative mitochondrial function (RMF). Value shown are the means of at least *n* = 3 independent samples ± the SD. *Significant differences (*p*≤0.05) with respect to the BD step.

Mitochondria are both the source of and the site for the detoxification of ROS in yeast. A physiological stimulus for ATP synthesis can become a pathological stimulus for ROS generation [13]. The STF2p protein may act as stabilising factor that enhances the inhibitory action of the Inh1p protein in the F1F0-ATP synthase; homodimers of the Inh1p protein bind directly to the F1-sector, allowing the maintenance of intracellular ATP levels [14]. Therefore, we evaluated whether changes in the regulation of ATP hydrolysis correlated with the enhancement of the cell dehydration tolerance. Both the Δ*inh1* and Δ*inh1*, *Galp-INH1* strains did not show improved survival relative to the reference strain (at ∼35%) (Fig. 4C). The increase in the cellular ATP level reduces the flux through the glycolytic pathway, thus inducing a reduction in pyruvate accumulation [15]. Figure 5 shows the evaluation of the cellular pyruvate and ATP concentrations for the Δ*stf2* and Δ*stf2*, *GALp-STF2* strains before cell dehydration (BD) and after the rehydration process (AR). The cells of the *BY4742*, *GALp* and Δ*stf2* strains showed similar patterns of ATP content BD and AR, increasing 4 fold and 2 fold, respectively. In the Δ*stf2*, *GALp-STF2* cells AR, less than 90% of the ATP level of the reference strain was observed (Fig. 5A). The mutated strains did not exhibit statistically significant changes in their pyruvate concentrations relative to the reference strain BD and AR (Fig. 5B). With regard to BD and AR, a minor discrepancy was observed between the mitochondrial mass and ΔΨ_m_ in the cells of the *BY4742*, *GALp*; Δ*stf2* and Δ*stf2*, *GALp-STF2* strains, but no significant change in their RMFs was observed (Fig. 5C), suggesting that variations in the mitochondrial function did not play a significant role in the cellular ATP content during the dehydration process. Therefore, we evaluated three petite mutant strains (*Δmtg1*, *Δmsh1*, and *Δnam1*) overexpressing the *STF2* gene to exclude the possibility that the increase in cell dehydration tolerance was a consequence of lack of synchronicity between ATP and pyruvate metabolism (Fig. 4C). Stationary cells from the Δ*mtg1*, *GAL_p_-STF2*; Δ*msh1*, *GAL_p_-STF2*; and Δ*nam1*, *GAL_p_-STF2* strains after galactose induction showed survival rates (∼75%) similar to that of the *Δstf2*, *GALp-STF2* strain (Fig. 1).

### Cells over-expressing STF2p show a reduction in DHE fluorescence after H_2_O_2_ stress

**Figure 6:**
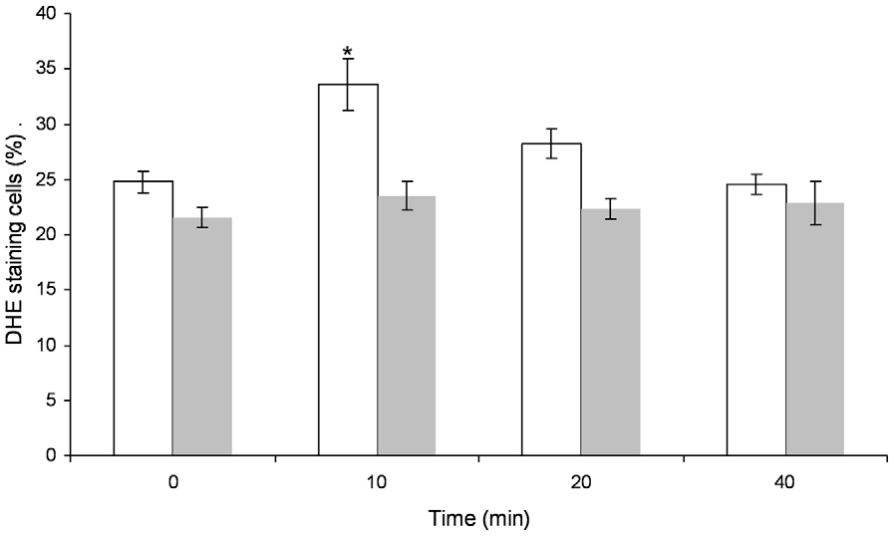
Levels of DHE accumulation after oxidative stress induced by H_2_O_2_. BY4742 (white bars) and *Δstf2*, *pGAL-STF2* (grey bars) cells were exposed to 4 mM H_2_O_2_, and at the indicated times, aliquots were collected to determine the number of DHE-positive cells. The represented data are the means of *n* = 6 determinations ± the SD. *Significant differences (*p*≤0.05) for each time with respect to the *Δstf2*, *pGAL-STF2* strain.

Stationary-state cells of the BY4742, *GALp* and Δ*stf2*, *GALp-STF2* strains were induced for 4 h with 2% galactose and exposed to 4 mM H_2_O_2_ (Fig. 6). The STF2p-over-expressing strain showed a reduction in the number of DHE-positive cells. As shown in Figure 6, after 10 min of H_2_O_2_ treatment, the percentage of cells accumulating ROS was ∼10% less of the value for the reference strain, supporting the hypothesis that STF2p acts as an antioxidant. However, after 20 min and 40 min of H_2_O_2_ stress, the number of DHE-positive cells did not exhibit significant differences, suggesting that STF2p does not have a strong positive effect on H_2_O_2_ clearance.

## Discussion

It has been reported previously that some highly hydrophilic proteins are commonly induced during water-deficit conditions [3]. Among the 12 *S. cerevisiae* hydrophilin proteins, we found that only STF2p, SIP18p, GRE1p, YJL144wp and NOP6p are necessary for the cells to overcome dehydration stress; only STF2p (involved in the regulation of the mitochondrial F1F0-ATP synthase) and NOP6p (an rRNA-binding protein required for 40S ribosomal subunit biogenesis) have known functions in yeast [16], [17]. The overexpression of the above proteins significantly enhanced cell viability under stress conditions. Therefore, considering the cell viability results and the apparently uncoupled cellular roles of the yeast STF2p, SIP18p, GRE1p, YJL144w, and NOP6p hydrophilins, we suggest that the roles as putative intracellular cell protectors might not be their only activity, as was shown for the group 3 late embryogenesis abundant (LEA) proteins, which prevent both protein aggregation and membrane fusion [18]. In this present study, we characterised the role of STF2p in dehydration stress and examined the possible physiological relationship between the overexpression of STF2p and the enhancement of viability after the induction of stress. The prevention of ROS accumulation in cells of the Δ*stf2*, *GALp-STF2* strain, during both the desiccation-rehydration process and H_2_O_2_ oxidative stress, indicates that STF2p is a protein with antioxidant capabilities, as has been reported for some plant LEA proteins [5]. Thus, the overexpression of STF2 prevents ROS accumulation and, consequently, cell apoptosis [19], [20]. The strains with highly significant viability rates, the GRE1p-, YJL144wp- and NOP6p-overexpressing strains, yielded viability and ROS results that were contrary to those for the Δ*stf2*, *GALp-STF2* strain. Perhaps, as was suggested for the LEA proteins, GRE1p, YJL144wp and NOP6p behave as molecular shields that prevent protein aggregation by steric or electrostatic repulsion, analogous to the polymer stabilisation of colloidal suspensions [21]; however, no direct evidence of functional mechanisms were described. It is well documented that mitochondrial function is necessary to maintain low intracellular ROS levels under both saline and osmotic stress conditions [22]. In addition, a physiological stimulus for ROS generation can become a stimulus for ATP synthesis in growing cells [13]. Most of the mechanisms of cellular tolerance to harsh conditions are driven via plasma membrane ATPase and vacuolar ATPase functions, processes that require large amounts of ATP to overcome acidic or osmotic stress; thus, a low ATP concentration could compromise cell viability during stress conditions [23], [24]. However, the accumulation of ATP reduces *S. cerevisiae* glycolytic activity, preventing pyruvate formation [15]. During the drying and rehydration process, the Δ*stf2*, *GALp-STF2* strain showed lower levels of accumulated ATP than the Δ*stf2* strain, and both strains had similar pyruvate concentrations after the stress induction, supporting the idea that the different viability values were not a consequence of achieving critical values for ATP and pyruvate. STF2p is a modulator of the INH1p regulatory peptide, which acts on the F_1_F_0_-ATP synthase complex [25], and the deletion or altered expression of the *INH1* gene could decrease the ATP supply or enhance cell growth and pyruvate production, respectively [26]. Considering the lack of correlation between the ATP and pyruvate concentrations in the *STF2* strains during the dehydration-rehydration process and the similar viabilities of the Δ*inh1*, *GALp-INH1* and Δ*inh1* strains, we conclude than the -lack of synchronicity between the glycolytic pathway and ATP synthesis did not have a major role in the improved survival rate of the *stf2*, *GALp-STF2* strain. Moreover, the overexpression of the *STF2* gene in the petite and non-petite strains resulted in similar viability rates. This result provides evidence that STF2p may allow the cell to survive by stabilising other cellular proteins rather than by interacting with apoptotic proteins, such as NUC1, shuttling from the mitochondria to the nucleus or by a reconfiguration of metabolism via the mitochondrial retrograde signal that is involved in nutrient sensing and cell aging [27], [28].

The present work provides evidence that STF2p allows yeast cells to survive during dehydration stress by contributing to the cellular antioxidant capacity that prevents ROS accumulation rather than by the inhibition of apoptotic proteins. Further studies will be necessary to establish the functional mechanisms of yeast hydrophilins which provide dehydration stress tolerance to the cells.

With recent advances in tissue engineering, cell transplantation and genetic technology, the successful long-term storage of living cells is of critical importance. Studies in yeast may provide a better understanding of desiccation-tolerance genetics for potential applications in biomedicine, plant biotechnology, and beverage and bio-ethanol technology.

## Materials and Methods

### Strains and plasmids

Table 1 summarises the yeast strains and plasmids used in this study. The single null mutant strains and the reference strain, all in the BY4742 genetic background, were purchased from EUROSCARF (Frankfurt, Germany). The yeast strain expressing the *GFP-STF2* chromosomal fusion was purchased from Invitrogen. Recombinant DNA techniques were performed according to standard protocols [29]. The synthetic genes (*GON7*, *GRE1*, *HSP12*, *INH1*, *NOP6*, *RPL42A*, *SIP18*, *STF2*, *WWM1*, *YBR016w*, *YJL144w*, and *YNL190w*) were obtained by PCR and cloned into the pGREG505 yeast expression vector (under the control of the *GAL1* promoter) digested with *Sal*I. The plasmids were then used to transform a yeast strain in which the corresponding gene had been deleted. The pGREG575 vector was used to express GFP-tagged proteins. Transformants were selected by plating on synthetic glucose media lacking leucine. Leu^+^ transformants were selected and re-streaked to obtain single colonies, which were confirmed by PCR using the primer pair GALFw and CYCRv (Table 2) and by testing for the loss of the *LEU* marker. The PCR fragments were obtained using BY4742 genomic DNA as a template together with the primer pairs shown in Table 2. The amplification reactions contained single-strength PCR buffer (Roche, Mannheim, Germany), 1.25 mM dNTPs, 1.0 mM MgCl_2_, 0.3 µM of each primer, 2 ng µl^−1^ template DNA and 3.5 U DNA polymerase (Roche) in a total volume of 100 µl. All of the reactions were carried out using a PCR Express thermal cycler for 15 cycles, as follows: denaturation, 2 min at 94°C; primer annealing, 30 s at 55°C; and primer extension, 1 min at 68°C.

**Table 1:** Plasmids and yeast strains used in this study.

*Strains*	Relevant characteristics	References
BY4742	MATα, *his3*Δ*1*, *leu2Δ0*, *lys2Δ0*, *ura3Δ0*	[33]
*STF2-GFP*	MATα, *his3*Δ*1*, leu2Δ*0*, *lys2*Δ*0*, *ura3*Δ*0*, *STF2-GFP-KanMX*	Invitrogene
Δ*stf2*	BY4742, *stf2::kanMX4*	EUROSCARF
Δ*sip18*	BY4742, *sip18::kanMX4*	EUROSCARF
Δ*hsp12*	BY4742, *hsp12::kanMX4*	EUROSCARF
Δ*YBR016w*	BY4742, *YBR016w::kanMX4*	EUROSCARF
Δ*wwm1*	BY4742, *wwm1::kanMX4*	EUROSCARF
Δ*gre1*	BY4742, *gre1::kanMX4*	EUROSCARF
Δ*YJL144w*	BY4742, *YJL144w::kanMX4*	EUROSCARF
Δ*nop6*	BY4742, *nop6::kanMX4*	EUROSCARF
Δ*gon7*	BY4742, *gon7::kanMX4*	EUROSCARF
Δ*YNL190w*	BY4742, *YNL190w::kanMX4*	EUROSCARF
Δ*inh1*	BY4742, *inh1::kanMX4*	EUROSCARF
BY4742, *GAL_p_*	BY4742+pGREG505Δh	This work
BY4742, *GAL_p_G*	BY4742+pGREG575Δh	This work
Δ*stf2*, *GAL_p_-STF2*	Δ*stf2*+pGREG505st	This work
Δ*stf2*, *GAL_p_G-STF2*	Δ*stf2*+pGREG575gst	This work
*Δsip18*, *GAL_p_-SIP18*	Δ*sip18*+pGREG505si	This work
Δ*hsp12*, *GAL_p_-HSP12*	Δ*hsp12*+pGREG505hs	This work
Δ*YBR016w*, *GAL_p_-YBR016w*	Δ*rif2*+pGREG505yb	This work
Δ*wwm1*, *GAL_p_-WWM1*	Δ*wwm1*+pGREG505ww	This work
BY4742, *GAL_p_-TIF11*	BY4742+pGREG505tf	This work
Δ*gre1*, *GAL_p_-GRE1*	Δ*gre1*+pGREG505gr	This work
Δ*YJL144w*, *GAL_p_-YJL144w*	Δ*gre1*+pGREG505yj	This work
Δ*nop6*, *GAL_p_-NOP6*	Δ*nop6*+pGREG505np	This work
Δ*gon7*, *GAL_p_-GON7*	Δ*gon7*+pGREG505gn	This work
Δ*gon7*, *GAL_p_-YNL190*	Δ*gon7*+pGREG505yl	This work
BY4742, *GAL_p_-RPL42A*	BY4742+pGREG505rp	This work
Δ*inh1*, *GAL_p_-INH1*	Δ*inh1*+pGREG505ih	This work
***Plasmids***		
pGREG505Δh	*GAL1_p_-Sal*I-*CYC1_t_*-*KanMX4*-*LEU2*-*bla*	[34]
pGREG575Δh	*GAL1_p_-GFP*-*Sal*I-*CYC1_t_*-*KanMX4*-*LEU2*-*bla*	[34]
pGREG505st	*GAL1_p_-STF2*-*CYC1_t_*-*KanMX4*-*LEU2*-*bla*	This work
pGREG575gst	*GAL1_p_-GFP-STF2*-*CYC1_t_*-*KanMX4*-*LEU2*-*bla*	This work
pGREG505si	*GAL1_p_-SIP18*-*CYC1_t_*-*KanMX4*-*LEU2*-*bla*	[34]
pGREG505hs	*GAL1_p_-HSP12*-*CYC1_t_*-*KanMX4*-*LEU2*-*bla*	This work
pGREG505yb	*GAL1_p_-YBR016*-*CYC1_t_*-*KanMX4*-*LEU2*-*bla*	This work
pGREG505ww	*GAL1_p_-WWM1*-*CYC1_t_*-*KanMX4*-*LEU2*-*bla*	This work
pGREG505tf	*GAL1_p_-TIF11*-*CYC1_t_*-*KanMX4*-*LEU2*-*bla*	This work
pGREG505gr	*GAL1_p_-GRE1*-*CYC1_t_*-*KanMX4*-*LEU2*-*bla*	This work
pGREG505yj	*GAL1_p_-YJL144w*-*CYC1_t_*-*KanMX4*-*LEU2*-*bla*	This work
pGREG505np	*GAL1_p_-NOP6*-*CYC1_t_*-*KanMX4*-*LEU2*-*bla*	This work
pGREG505gn	*GAL1_p_-GON7*-*CYC1_t_*-*KanMX4*-*LEU2*-*bla*	This work
pGREG505yl	*GAL1_p_-YNL190*-*CYC1_t_*-*KanMX4*-*LEU2*-*bla*	This work
pGREG505rp	*GAL1_p_-RPL42A*-*CYC1_t_*-*KanMX4*-*LEU2*-*bla*	This work
pGREG505ih	*GAL1_p_-INH1*-*CYC1_t_*-*KanMX4*-*LEU2*-*bla*	This work

**Table 2:** Primers designed for PCR.

Primer	Oligonucleotide sequence^a^
STF2Fw	5′-**GAATTCGATATCAAGCTTATCGATACCGTCGACA**TGACGAGAACAAACAAG-3′
STF2Rv	5′-**GCGTGACATAACTAATTACATGACTCGAGGTCGAC**TCATTCCTTTTGGACGT-3′
HSP12Fw	5′-**GAATTCGATATCAAGCTTATCGATACCGTCGACA**TGTCTGACGCAGGTAG-3′
HSP12Rv	5′-**GCGTGACATAACTAATTACATGACTCGAGGTCGAC**TTACTTCTTGGTTGGGTC-3′
YBRFw	5′-**GAATTCGATATCAAGCTTATCGATACCGTCGACA**TGTCTGCTAACGATTAC-3′
YBRRv	5′-**GCGTGACATAACTAATTACATGACTCGAGGTCGAC**TTAGAATAGCATATCCATG-3′
WWM1Fw	5′-**GAATTCGATATCAAGCTTATCGATACCGTCGACA**TGGCTCAAAGTAAAAGTAAT-3′
WWM1Rv	5′-**GCGTGACATAACTAATTACATGACTCGAGGTCGAC**CCATGGATATGCTATTCTAA-3′
TIF11Fw	5′-**GAATTCGATATCAAGCTTATCGATACCGTCGACA**TGGGTAAGAAAAACAC-3′
TIF11Rv	5′-**GCGTGACATAACTAATTACATGACTCGAGGTCGAC**TTAAATGTCATCAATATC-3′
GRE1Fw	5′-**GAATTCGATATCAAGCTTATCGATACCGTCGACA**TGTCCAATCTATTAAACAAG-3′
GRE1Rv	5′-**GCGTGACATAACTAATTACATGACTCGAGGTCGAC**CTACCAGACGCCTTG-3′
YJL144wFw	5′-**GAATTCGATATCAAGCTTATCGATACCGTCGACA**TGTTAAGGAGGGAAACTT-3′
YJL144wRv	5′-**GCGTGACATAACTAATTACATGACTCGAGGTCGAC**TTATCATGAACAACGGCGAG-3′
NOP6Fw	5′-**GAATTCGATATCAAGCTTATCGATACCGTCGACA**TGGGGTCCGAGGAAG-3′
NOP6Rv	5′-**GCGTGACATAACTAATTACATGACTCGAGGTCGAC**TCATTTAAGTAGTTTGGCT-3′
GON7Fw	5′-**GAATTCGATATCAAGCTTATCGATACCGTCGACA**TGAAACTACCGGTAGC-3′
GON7Rv	5′-**GCGTGACATAACTAATTACATGACTCGAGGTCGAC**TAAACAGCATCTTCGTC-3′C
YNL190wFw	5′-**GAATTCGATATCAAGCTTATCGATACCGTCGACA**TGAAGTTCTCTTCTGTTA-3′C
YNL190wRv	5′-**GCGTGACATAACTAATTACATGACTCGAGGTCGAC**TTATAATAGTAATAAGGCACC-3′
RPL42Fw	5′-**GAATTCGATATCAAGCTTATCGATACCGTCGACA**TGGGTATGTTATAACC-3′
RPL42wRv	5′-**GCGTGACATAACTAATTACATGACTCGAGGTCGAC**TCAGAATTGCAAAGCTTGAC-3′
INH1Fw	5′-**GAATTCGATATCAAGCTTATCGATACCGTCGACA**TGTTACCACGTTCAGC-3′
INH1Rv	5′-**GCGTGACATAACTAATTACATGACTCGAGGTCGAC**TTATTTGGTCATCGAGTC-3′
GALFw	5′-GAAAAAACCCCGGATTCTAG-3′
CYCRv	5′-ATAACTAATTACATGACTCGAG-3′

### Growth conditions and desiccation-rehydration process

Yeast strains were grown in shake flasks at 150 rpm in SC medium containing 0.17% yeast nitrogen base (Difco), 2% glucose, 0.5% (NH_4_)_2_SO_4_, 25 mg·l^−1^ uracil, and 42 mg·l^−1^ lysine and histidine. The desiccation-rehydration process and yeast viability determinations were performed as described previously [30].

### Tests for apoptotic markers

The DHE staining, Annexin V/PI co-staining and TUNEL staining were performed as described in [31]. The same samples were analysed by fluorescence microscopy. To determine the frequencies of the morphological phenotypes revealed by the TUNEL, DHE and Annexin V/PI staining, at least 10^6^ cells from three independent experiments were evaluated using flow cytometry and FloMax software (Partec GmbH, Germany).

### Microscopy

Cultures of strains harbouring the *GFP*-tagged genes were grown to the stationary phase in SC medium. The cells were washed with 1× PBS buffer (pH 7.4) and fixed with 70% ethanol for 10 min at R.T. Fluorescence was viewed using a Leica fluorescence microscope (DM4000B, Germany). A digital camera (Leica DFC300FX) and the Leica IM50 software were used for the image acquisition. Confocal images were obtained using a laser microscope (Nikon TE2000-E) equipped with a digital camera (Nikon DXM1200C), and overlaid images using NIS-Elements software (Nikon).

### Determination of ATP and pyruvate concentrations

The cellular pyruvate concentration was determined using the Pyruvate Assay Kit (BioVision Research Products, USA), and the ATP content was assessed with the ATP Bioluminescence Assay Kit HS II (Roche Applied Science, Germany). The quantification was carried out using a POLARstar Omega microplate reader equipped with two reagent injectors (BMG LABTECH, USA).

### Assessment of mitochondrial changes

The changes in mitochondrial mass and ΔΨ_m_ were assessed using JC-1 (Molecular Probes Inc.) as previously described [32]. JC-1 allows the simultaneous quantification by flow cytometry of both the mitochondrial mass (green fluorescence) and ΔΨ_m_ (red fluorescence). We defined the relative mitochondrial function (RMF) as the ratio of JC-1 red∶green, which reflects the changes in ΔΨ_m_ per unit mitochondrial mass.

### Statistical analysis

The results were statistically analysed by one-way ANOVA and the Scheffé test using the SPSS 15.1 statistical software package. The statistical significance were set at *P*≤0.05 and *P*≤0.01.
